# Accommodating conscientious objection in the midwifery workforce: a ratio-data analysis of midwives, birth and late abortions in 18 European countries in 2016

**DOI:** 10.1186/s12960-020-00482-y

**Published:** 2020-06-08

**Authors:** Valerie Fleming, Clare Maxwell, Beate Ramsayer

**Affiliations:** Faculty of Health, Liverpool John Moores University, 16-19 Webster St, Liverpool, L3 2ET UK

**Keywords:** Abortion, Conscientious objection, Abortion services, Midwifery, Europe

## Abstract

**Background:**

In recent years, the role of a midwife has expanded to include the provision of abortion-related care. The laws on abortion in many European countries allow for those who hold a conscientious objection to participating to refrain from such participation. However, some writers have expressed concerns that this may have a detrimental effect on the workforce and limit women’s access to the service.

**Method:**

The aim of this study was to provide a picture of the potential exposure midwives in Europe have to late abortions, an important factor in the integration of accommodation of conscientious objection to abortion by midwives into workload planning. We collected data from Ministries of Health or government statistical departments in 32 European countries on numbers of births, abortions, late abortions and midwives in 2016. We conducted a ratio-data analysis in those countries that met the inclusion criteria.

**Results:**

Eighteen of the 32 countries provided full data; thus, our calculations are based on a total of 4 036 633 live births, 49 834 late abortions and a total of 132 071 midwives. The calculated ratios of live births to midwife, abortions to midwife and late abortions to midwife illustrate the wide variations between countries in relation to ratios of midwives to live births (15.22–53.99) and late abortions (0.17–1.47)

**Conclusions:**

This study provides the first comprehensive insight to ratios relating to birth and abortion, especially late abortion services, with regard to the midwifery workforce. It is essential to improve the reporting of abortion data and access to it within Europe to support evidence-informed decisions on optimising the contribution of the midwifery workforce especially within highly contentious fields such as abortion services. The study’s findings suggest that there should be neither be any difficulty for those who are responsible for workload allocation nor compromises to a women’s right to abortion services.

## Background

Midwives constitute a valuable resource in health services globally by providing care to women throughout the pregnancy continuum [1]. In Europe, midwifery care, however, is varied in its scope of practice and the quality of its provision [2]. A number of definitions of the midwife also exist, with the World Health Organization’s (WHO) [3] now subtly differing from that of the International Confederation of Midwives (ICM) [4]. Both, however, remain clearly focused on the provision of care during a normal pregnancy with the ICM noting that the midwife’s role may extend to sexual and reproductive care. A major break from these definitions occurred in the “State of the World’s Midwifery Report” [5] in which midwifery is defined as:

the health services and health workforce needed to support and care for women and newborns, including sexual and reproductive health and especially pregnancy, labour and postnatal care. This includes a full package of sexual and reproductive health services, including preventing mother-to-child transmission of HIV, preventing and treating sexually transmitted infections and HIV, preventing pregnancy, dealing with the consequences of unsafe abortion and providing safe abortion in circumstances where it is not against the law. (pp.3-4)

The UNFPA definition clearly moves the midwife into a different role but one which is supported by other UN agencies such as the WHO. Such a move has come about in response to the numbers of women dying from unsafe abortions in countries where legal abortion is not possible. In Europe, however, from the latter half of the 20th century, most countries enacted laws permitting abortion to some extent [6]. The only European countries in which abortion is still forbidden, except when there is a threat to the woman’s life, are Liechtenstein and Malta, each of which punishes both the woman and the involved health professionals by a period of imprisonment [7, 8]. Conversely, many European countries permit abortion on the request of the women in the first trimester of pregnancy, while others require medical authorisation from the outset. Despite legislation, abortion remains a very volatile subject, with arguments highly polarised [9], the topic often being portrayed in the media as pro-life vs pro-choice or even rampant feminism vs religious fanaticism [10]. This has the potential to cause much pain and controversy for both service users and providers. One of the major arguments in the present time, rather than concerning the right of women to have abortions, is about the rights of health professionals to object on conscience grounds to providing abortion services [9], a key issue being that this could create imbalances in the workforce. Such arguments exist despite most countries’ laws including this right and conscience, as a core element of human rights, being protected in documents such as the European Convention on Human Rights [11]. According to the latest figures from the WHO, of the 30 European countries that permitted abortion at the time of data collection, 25 included a so-called conscience clause permitting health care providers, who hold a legitimate objection, to desist from participating in the provision of abortion [12].

Most of the above arguments are specific to abortion and apply to all relevant health professionals. However, in recent times, it is often midwives who are at the centre of controversies relating to conscientious objection.

### Abortion and conscientious objection

In the United Kingdom (UK), two senior midwives lost a Supreme Court case which ruled that conscientious objection must be restricted to “hands on” activities [13]. Similar cases affecting midwives have also been reported in other European countries notably Croatia and Sweden, the latter of which does not have a legal position on conscientious objection. One of the midwives who lost her case in Sweden has lodged it with the European Court of Human Rights [14].

The potential workforce issues resulting from midwives who make conscientious objections have never been addressed with a quantitative focus on the associated workload. Having evidence-based information however is essential for a better understanding of and contribution to the debate.

This article therefore presents an analysis of relevant statistics in 18 European countries in relation to potential exposure to late abortions by midwives in order to throw new light on the controversial topic in relation to the midwifery workforce.

The literature is vague as to the extent to which conscientious objection should be permitted. Taking note of the ambiguities, the authors of one article propose that “European countries should critically assess the laws governing conscientious objection and its effects on women’s rights” ([15], p. 231).

This position has been supported by writers who suggest that the various treaties permitting conscientious objection on the grounds of human rights have compromised women’s right to abortions [16]. However, in a case study report on four European countries, the conclusion drawn is that, although complex, it is possible to accommodate individuals who object to providing abortion-related care, while still ensuring that women have access to legal health care services in the countries concerned [17].

The academic commentaries on conscientious objection also are divided. The seminal work of Wicclair provided a comprehensive link between conscience and integrity in medicine, concluding that *carte blanche* rights of conscientious objection should not be given but rather that respect for moral integrity of the physician, even in practices endorsed by the medical profession, is the best way forward [18]. Claims of conscientious objection thus should derive from the importance attributed to the integrity underpinning them. Weinstock (p. 12) comments that when a health professional’s right to conscientious objection is observed, “respect [is afforded to] the moral agency of those who hold reasonable dissenting views” [19]. In the same vein, Curlin et al. ([20], p. 1891) reflected that “acting conscientiously is the heart of the ethical life” hypothesising that if medical practitioners give this up they no longer have the capacity to make moral judgments or act in accordance with them.

Other writers challenge such positions proposing that the rights of health care professionals to allow their private values should not interfere with their work [21]. Conscientious objection to abortion-related care has also been labelled as “dishonourable disobedience” ([22], p .12). Conversely, Pellegrino avers that a health professional’s conscience or religious values must never be placed in a position secondary to the health service’s requirements [23]. Taking a nuanced approach, Neal [24] suggests that the apparent expansion of conscientious objection claims is based on poorly defined or even contradictory professional guidelines and there is a need for sound research establishing working definitions.

Various professional bodies or regulatory authorities have established such guidelines, the International Federation of Obstetricians and Gynaecologists’ (FIGO) criteria for conscientious objection [25] being to provide notice, refer patients timeously and provide emergency care. While brief and practical, the standards, like those of other such bodies, are not based on research with practitioners. A “White Paper” drawing on international, multidisciplinary literature sums up the issue and develops a road map for the future [26]. The authors point out the lack of well carried out empirical research on the topic but conclude from reviewing the available evidence that there is a growing trend towards refusal to provide certain reproductive health services, especially abortion. Acknowledging the difficulty of the situation, they recommend that a standard definition of conscientious objection be developed together with accompanying obligations.

### Role of midwives

What is evident from the literature is that its key focus is on medical practitioners, and if midwives are mentioned, it is in a secondary position [9] despite the WHO’s emphasis on them as key providers of abortion services [27]. This has most recently been discussed in two articles, one specifically focusing on abortion [9] and the other on end of life care [28], each of which urged nurses and midwives respectively to be more proactive in contributing to the debate.

With the overwhelming change from surgical to medical abortions in both first and second trimesters of pregnancy, there is an increasing role for midwives and the above arguments are clearly relevant to them. In the past, abortions were carried out surgically exclusively by doctors who may have been assisted by nurses or midwives in an operating theatre. Both in the theatre and pre-and post-operatively, those who expressed a conscientious objection to the procedure were not expected to participate. With medical abortions, the prescription is generally written by a medical practitioner, but the drug is often administered by a nurse, midwife or the woman herself. While in the first trimester in some countries the woman now may labour at home, in the second trimester in most countries, she is cared for throughout the subsequent labour in an inpatient setting by midwives, or occasionally, nurses, thereby increasing their workload.

Until recently, only one article acknowledged this, commenting that many more health professionals are now involved over a much longer period of time [29]. However, in the last 2 years, the topic of midwives or nurses in relation to conscientious objection has come to the fore with two articles emphasising their invisibility [9, 28].

Taking into account the divergence of opinions shown by both academic writers and policy makers and the lack of visibility of midwives, we believe that the whole debate on conscientious objection would benefit from giving consideration to numerical data, not simply considering the numbers of abortions but their relationship to other variables.

We have chosen to focus on midwives in relation to abortions conducted after the first trimester of pregnancy, because it is mainly midwives who are expected to participate in drug administration and the subsequent care for women in labour, including delivery of the foetus and placenta and the provision of immediate postnatal care [27].

This background has shown that a controversial debate around conscientious abortion exists and that both philosophical arguments and laws exist for the provision and the opportunity to conscientiously object to the provision of abortion services. There is, however, a lack of quantitative research showing the dimensions of the topic, and so accurate workload indices cannot be produced. This study seeks to address the issue.

## Methods

### Aim

The aim of this study was to provide a picture of the potential exposure midwives in Europe have to abortions conducted after the first trimester of pregnancy (hereafter “late abortions”), an important factor in the integration of accommodation of conscientious objection to abortion by midwives into workload planning. This has been investigated by collecting and analysing data on late abortions, live births and numbers of midwives by European country to provide a ratio of late abortions to midwife.

### Sample and inclusion/exclusion criteria

We selected 32 European countries due to 28 of them being Member States of the European Union (EU) during the period of analysis and the remaining four being signatories to the Shengan accord, which provides for free movement without border controls between them. Of the 28 EU Member States, Croatia, Ireland and the United Kingdom (UK) were not part of the Shengan area.

We decided only to include and analyse robust statistical data provided by Ministries of Health or government statistics departments, with data provided by “third parties” excluded. Only countries that could provide full data sets on all variables were included in the analysis, with countries that had only partial or no data excluded.

### Data collection

A rigorous process of data collection was conducted between January 2018 and March 2019.

Data were collected for the four variables: “number of live births”, “number of abortions”, “number of late abortions” and “number of midwives according to the OECD [30]” for the year 2016, as this year offered the most complete data. This was not a straightforward process and a number of “stages” were undertaken (detailed below) in order to collect data that were considered robust and that met the inclusion criteria.

- ➢ Initially, we accessed the homepages of MoHs and government statistics departments of every country for data on the four variables.
- ➢ Next, due to countries reporting differently on health-related figures and, in order to deal proactively with expected reporting problems, we contacted each MoH by email requesting data on the four variables. Discrepancies were resolved by returning to the original data and questioning with MoH how they were categorised. In one case (Slovenia), the number of midwives was such an outlier that we returned to the Ministry and were told that only the midwives with a degree were included in their calculations. Midwives with a lower academic qualification were also in practice, so to obtain their numbers, they referred us to the midwifery organisation whose official confirmed the MoH’s position and supplied the remaining numbers. We then returned to other countries to see if this was a problem elsewhere and it was not.
- ➢ If the first two approaches did not provide the required data, we contacted the MoHs concerned personally by phone to request the data.
- ➢ When countries provided health reports in languages that we could not understand, we contacted researchers from the countries concerned and asked them to translate relevant passages and to identify relevant data. Full data sources are provided in the accompanying notes.

We identified differences related to the reporting of abortions within the sample. Several countries’ abortion data, most noticeably those in Eastern Europe, contained data on “spontaneous abortions” as well as “induced” and “other” abortions. On inquiring with the MoHs concerned, a comprehensive response was obtained from Latvia which stated “other” meant “abortion with unknown origin or termination of the pregnancy where the factors that lead to this termination are unknown” [personal communication with the Centre for Disease Prevention and Control of Latvia to the authors, 28 September 2018]. Similar confirmations were then received from the other countries. As it was thus clear that these figures were active terminations rather than miscarriages, they have been included in our analysis among the number of abortions. Abortions classified as “spontaneous” conversely have been omitted from our analyses.

### Data analysis

All our data were inputted into Excel by one author and double-checked for discrepancies by another author and a statistician against the raw data. (The sources of all data are available on request.) We conducted a ratio data analysis among those countries that met the inclusion criteria. Ratio distributions were calculated between midwives to live births, late abortions to live births and late abortions to number of midwives for each country using Excel. We created box plots using Excel in relation to live births and late abortions/midwife, calculating the median, range and the highest and lowest ratios. We also created a line comparison graph using Excel to show live births and late abortions per midwife by country.

### Ethics approval

As our study is based on published or freely available statistical data and no data were sought from individuals, no approval from an ethics committee was required.

## Results

Table 1 displays the data for the number of midwives, live births, total abortions and late abortions for all of the 32 countries.

**Table 1:** Midwives, live births, abortions and late abortions in 2016

Country	Midwives	Live births	Total abortions	Late abortions
**Austria**	1478	87 675		
**Belgium**	3263	121 161	21 900	
**Bulgaria**	3254	64 984	27 782	
**Croatia**	1698	37 537	5960	
**Cyprus**	268	9455		
**Czech Republic**	3904	112 663	20 409	935
**Denmark**	1873	61 614	3162	
**Estonia**	440	14 003	4323	97
**Finland**	2283	52 814	9665	397
**France**	22 761	783 640	168 519	6192
**Germany**	23 000	792 495	98 721	3850
**Greece**	2701	92 898		
**Hungary**	1628	93 063	30 439	
**Iceland**	265	4034	1044	50
**Ireland**	2085	63 897		
**Italy**	16 507	437 438	84 874	3366
**Latvia**	403	21 759	4366	265
**Lithuania**	921	30 623	4502	164
**Liechtenstein**	10	378		
**Luxembourg**	211	6050	580	
**Malta**	217	4227		
**Netherlands**	3778	172 520	30 144	5538
**Norway**	2943	58 890	12 733	546
**Poland**	22 464	382 257	1098	
**Portugal**	2548	87 126	15 959	543
**Romania**	3337	188 415	63 518	
**Slovakia**	1795	57 557	15 277	1500
**Slovenia**	779	20 345	3736	201
**Spain**	8531	410 583	93 131	5749
**Sweden**	7303	117 425	38 143	2431
**Switzerland**	2593	87 883	10 256	513
**UK**	31 317	774 835	202 469	17 497
**Total**	176 558	5 250 244	972 710	49 834

Table 1 shows that 18 of the 32 European countries provided data on the four variables we analysed in our study (see Table 2). A further eight European countries provided data concerning numbers of births and abortions but no information on late abortions conducted after 12 weeks of gestational age. Those were Belgium, Bulgaria, Croatia, Denmark, Hungary, Luxembourg, Poland and Romania. Finally, six European countries provided data on births and midwives but not on abortions, or the latter had only been estimated by third parties which did not meet our inclusion criteria. Those were Austria, Cyprus, Greece, Ireland, Liechtenstein and Malta.

**Table 2:** Midwife, live birth and late abortion and ratios in countries with all data in 2016

Country	Midwives	Live births	Total abortions	Late abortions	Late abortions to live births	Live births to midwife	late abortions: midwife
**Czech Republic**	3904	112 663	20 409	935	0.008	28.86	0.24
**Estonia**	440	14 003	4323	97	0.007	31.83	0.22
**Finland**	2283	52 814	9665	397	0.008	23.13	0.17
**France**	22 761	783 640	168 519	6192	0.008	34.43	0.27
**Germany**	23 000	792 495	98 721	3850	0.005	34.46	0.17
**Iceland**	265	4034	1044	50	0.012	15.22	0.19
**Italy**	16 507	437 438	84 874	3366	0.008	26.50	0.20
**Latvia**	403	21 759	4366	265	0.012	53.99	0.66
**Lithuania**	921	30 623	4502	164	0.005	33.25	0.18
**Netherlands**	3778	172 520	30 144	5538	0.032	45.66	1.47
**Norway**	2943	58 890	12 733	546	0.009	20.01	0.19
**Portugal**	2548	87 126	15 959	543	0.006	34.19	0.21
**Slovakia**	1795	57 557	15 277	1500	0.026	32.07	0.84
**Slovenia**	779	20 345	3736	201	0.010	26.12	0.26
**Spain**	8531	410 583	93 131	5749	0.014	48.13	0.67
**Sweden**	7303	117 425	38 143	2431	0.021	16.08	0.33
**Switzerland**	2593	87 883	10 256	513	0.006	33.89	0.20
**UK**	31 317	774 835	202 469	17 497	0.023	24.74	0.56
**Total**	132 071	4 036 633	818 271	49 834			

**Fig. 1:**
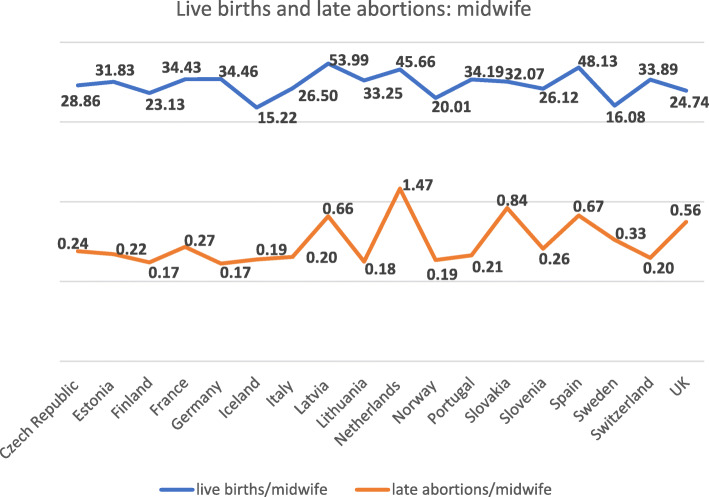
Details of live births and late abortions/midwife in 18 European countries in 2016

Table 2 shows that our calculations are based on a total of 4 036 633 live births, 49 834 late abortions and 132 071 midwives. In total, 818 271 abortions, including late abortions, were reported in those 18 countries. Table 2 displays the calculated ratios of live births to midwife, abortions to midwife and late abortions to midwife by the 18 countries that met the study inclusion criteria. This illustrates the wide variations between countries in relation to ratios of midwives to live births and late abortions, and abortions to live births. For example as shown in Fig. 1, the ratio of live births to midwife ranged from 15.22 in Iceland to 53.99 in Latvia and the ratio of late abortions to live births ranged from 0.05 in Germany to 0.32 in the Netherlands. In addition, the ratio of late abortions to midwife ranged from 0.17 in Germany and Finland to 1.47 in the Netherlands.

**Fig. 2:**
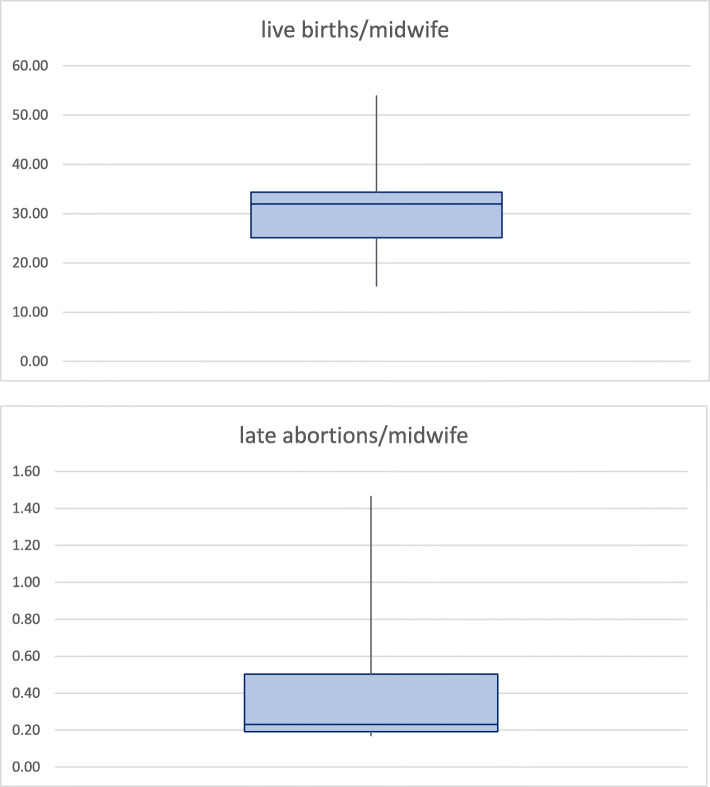
Ratio of live births and late abortions/midwife in 2016

Although our results in Table 2 show that there is a wide range in ratios of midwives to live births across the 18 countries, our calculated box plots in Fig. 2 show that the majority of midwives have a narrower range of calculated ratio of live births of between 25 and 35, with a median of 32. Similarly, although there is a wide variation in range of ratios of midwife to late abortions shown in Table 2, our calculated box plots in Fig. 1 show that the majority of midwives have a ratio 0.2 to 0.5, with a median of 0.22.

## Discussion

This first approach to quantifying the ratio of midwives to late abortions throughout Europe offers some interesting insights into the potential exposure midwives have to late abortion, which can be used to further the debate on accommodating conscientious objection to abortion by midwives in the workforce. Our analysis of late abortions is based on robust statistical data that were made available in 18 out of the 32 European countries. The comparisons we have drawn have not been previously published in a comprehensive report; thus, this paper provides a unique contribution concerning births and late abortions in relation to the midwifery workforce across Europe.

Table 1 shows that 5 250 066 live births took place in Europe in 2016. Our analysis is based on 4 036 633 live births taking place in the 18 countries which provided complete data sets and accounts for approximately 76.9% of all births that occurred in Europe in the year 2016. However, due to different underlying laws and conscience clauses, it is unlikely that this figure can be extrapolated to the figures to the percentage of abortions or late abortions. We rather interpret this figure of 76.9% in a way that it shows we achieved an inclusion of relevant European countries in our analysis, which represent over three quarters of all live births in Europe, despite having excluded 14 countries. Similarly, midwife numbers for the 18 countries amount to 176 558 which accounts for 74.9% of midwives in Europe, although it is acknowledged that there may be differences in how practising midwife numbers are recorded.

Our data show that the proportion of late abortions in relation to the number of births is low, even if the numbers of late abortions might sometimes be under reported. Our results show within Tables 1 and 2 consistently low ratios of late abortions to births and midwives which are further illustrated in Fig. 2 in the low median and narrow range for the majority of midwives in relation to these ratios. They add another dimension to the argument of Chavkin et al. [26] who concluded that their “best case’ studies [of four European countries] illustrate that it is possible to permit CO to abortion and still ensure that women have access to care” (p. 66). They also noted that conscientious objection to abortion presents a challenge to governments which may have to negotiate competing belief systems. In particular they highlight the rival rights and obligations in societies which are no longer theocratically centred so blurring lines between religious and political based conscience. Our study, unlike theirs, did not look at the underlying factors in the countries which we explored but rather presents a factual analysis of the numbers involved.

Our study shows that the potential exposure to late abortions by midwives is extremely low throughout the 18 countries and unlikely to affect workforce planning. Therefore, we query the claims of Heino et al. [15] and Zampas [16] that the increasing numbers of objectors are endangering abortion provision. We clearly show that the numbers of late abortions that may be expected to be encountered by midwives in the countries we included are negligible, even in the Netherlands, the country with the highest ratio in our analysis between late abortions and midwives. This also supports Wicclair´s stance, suggesting that “moral space” [18] or “discretionary space” [31] is also needed for midwives, in which they can practise without professional detriment and Sulmasy’s ([31], p. 29) argument that “a plural, liberal democratic society needs to foster the independence of its profession if it is to flourish”.

Although not a surprising finding, it should be noted that during the undertaking of this study discrepancies in the reporting of abortion data across Europe were prominent. Furthermore, the actual retrieval of data was challenging and time consuming and required an amount of persistence on the part of the research team. Although the authors of this study did not set out to analyse the accuracy of European abortion data, it is clear that a more transparent and streamlined mechanism of data reporting is needed in order to make the process of collecting and extrapolating data to various contexts such as conscientious objection to abortion less problematic.

### Strengths and limitations of the study

This study has several strengths and limitations. A major strength is that a robust and comprehensive process of data collection was undertaken, with the data provided by the MoHs throughout Europe enabling us to analyse a considerable amount of data which has not been undertaken previously. However, despite the transparency throughout Europe regarding the reporting of birth rates, the same could not be said for the reporting of abortions. The reporting of abortion figures and especially the reporting of late abortions differed with some countries providing no data on the gestational age when the abortion was conducted. It may be that the actual number of abortions carried out differs from what is reported in the official statistics of some countries with some abortions being reported as “curettage” or similar. It is further accepted that not every midwife included in the numbers in this study will be practising. Of those who are in current practice, some will not encounter women having late abortions as these often take place in specialised foetal medicine units. These limitations also apply to the ratio of midwives to births as again many midwives do not work in labour and delivery units. Thus, we would conclude that our findings concerning the ratio of midwives to late abortions are general in nature rather than context specific. However, this limitation is acknowledged and influences our conclusions. Additionally, we do not know if midwives might object in some cases but not all, and we have been unable to identify a database in which such information could be found. The method of data collection that we used also exhibits limitations, due to potential under-reporting of abortions by some countries. In addition, it might be that some health-care systems offer abortion services in private clinics that do not provide data on governmental databases, and as such, data on late abortions and midwives who work in such settings will not have been included in this study.

## Conclusions

This study provides the first comprehensive insight to ratios relating to birth and abortion, especially late abortion services, with regard to the midwifery workforce. While traditionally midwives have accompanied women during pregnancy, birth and the postnatal period, their role is now being extended to their becoming key personnel in the provision of abortion services.

Research related to midwifery workforce and abortion services is highly relevant for health-care systems because both the provision of and objection to those services are discussed and practised. Based on the identified limitations in our study that the reporting of abortion figures and especially the reporting of late abortions differed, we conclude that it is vital to improve the reporting of abortion data and access to it within Europe. We consider this necessary for conducting research that supports evidence-informed decisions on optimising the contribution of both nursing and midwifery workforces, especially within highly contentious fields of such as conscientious objection to abortion services.

We further conclude that quantitative data analysis contributes to clarification within the debate around CO. The data we have obtained now will therefore form the basis of a new database which we intend to update each year. This will enable us and other researchers to make some comparisons. Based on our findings, we reach the conclusion that comparability of the vital issues of abortions and births within European countries would be improved if both rates were reported consistently and transparently by all countries. Such reporting may lead to further understanding of similarities and differences in the women’s reproductive health arena in Europe. Therefore, we recommend the provision of reliable, detailed annual abortion statistics in all European countries.

Finally, there should be neither be any difficulty for those who are responsible for workload allocation nor compromises to a women’s right to abortion services. Given that relatively few midwives decide to make a conscientious objection to the provision of abortion services, and with knowledge of which midwives are conscientious objectors and sensible rostering, it should not be difficult to accommodate them without disadvantaging other non-objecting midwives or women´s access to abortion services.

## Data Availability

The datasets used and/or analysed during the current study are available from the corresponding author on reasonable request.

## Abbreviations

FIGO: International Confederation of Obstetricians and Gynaecologists
ICM: International Confederation of Midwives
MoH: Ministry of Health
WHO: World Health Organization
